# Blood-tumor barrier opening by MRI-guided transcranial focused ultrasound in a preclinical breast cancer brain metastasis model improves efficacy of combinatorial chemotherapy

**DOI:** 10.3389/fonc.2023.1104594

**Published:** 2023-02-10

**Authors:** Tasneem A. Arsiwala, Kathryn E. Blethen, Cullen P. Wolford, Dhruvi M. Panchal, Samuel A. Sprowls, Ross A. Fladeland, Brooke N. Kielkowski, Trenton A. Pritt, Peng Wang, Olivia Wilson, Jeffrey S. Carpenter, Victor Finomore, Ali Rezai, Paul R. Lockman

**Affiliations:** ^1^ Department of Pharmaceutical Sciences, School of Pharmacy, West Virginia University, Morgantown, WV, United States; ^2^ Department of Cardiovascular and Metabolic Sciences, Lerner Research Institute, Cleveland Clinic, Cleveland, OH, United States; ^3^ Rockefeller Neuroscience Institute, West Virginia University, Morgantown, WV, United States; ^4^ Departments of Neuroscience, Neuroradiology, and Neurosurgery, West Virginia University, Morgantown, WV, United States

**Keywords:** blood-brain barrer, blood-tumor barrier (BTB), focused ultrasound (MRgFUS), chemotherapeutic responses, drug delivery, efficacy, combinatorial therapeutic regime

## Abstract

Patients with metastatic breast cancer have high and continually increasing rates of brain metastases. During the course of the disease, brain metastases can occur in up to 30% of these patients. In most cases, brain metastases are diagnosed after significant disease progression. The blood-tumor barrier increases the difficulty of treating brain metastasis by preventing accumulation of chemotherapy within metastases at therapeutically effective concentrations. Traditional therapies, such as surgical resection, radiotherapy, and chemotherapy, have poor efficacy, as reflected by a low median survival rate of 5-8% after post-diagnosis. Low-intensity focused ultrasound (LiFUS) is a new treatment for enhancing drug accumulation within the brain and brain malignancies. In this study, we elucidate the effect of clinical LiFUS combined with chemotherapy on tumor survival and progression in a preclinical model of triple-negative breast cancer metastasis to the brain. LiFUS significantly increased the tumor accumulation of 14C-AIB and Texas Red compared to controls (p< 0.01). LiFUS-mediated opening of the BTB is size-dependent, which is consistent with our previous studies. Mice receiving LiFUS with combinatorial Doxil and paclitaxel showed a significant increase in median survival (60 days) compared to other groups. LiFUS plus combinatorial chemotherapy of paclitaxel and Doxil also showed the slowest progression of tumor burden compared to chemotherapy alone or individual chemotherapy and LiFUS combinations. This study shows that combining LiFUS with timed combinatorial chemotherapeutic treatment is a potential strategy for improving drug delivery to brain metastases.

## Introduction

Breast cancer is a significant public health concern for women, with nearly one out of every eight women expected to be diagnosed at some point in their lives in the United States (1). Although lung, liver, and bone metastases are more common, patients with brain metastases have the worst overall and breast cancer-specific survival (2, 3). In most cases, the prognosis and metastatic potential of breast cancer are determined by its molecular subtype. In patients with advanced peripheral disease, the incidence of central nervous system (CNS) metastases of the triple-negative subtype of breast cancer (TNBC) has increased to nearly 46 percent over the past few years (4). These patients have a poor prognosis, with a median survival of 5-7 months from initial diagnosis of the metastatic TNBC (5). While several therapeutic interventions are available for the peripheral disease, there are currently no TNBC-specific treatments for brain metastasis (6).

Brain metastases are currently treated with a combination of surgical resection, radiation, and or systemic chemotherapy (7). Whole-brain or stereotactic radiotherapy post-tumor resection reduces the risk of local recurrence and new metastasis formation (8, 9). This strategy may be difficult in cases of advanced TNBC brain metastasis due to the limited number of contrast-enhancing metastases, risk of radiation necrosis, radiation-induced neurological toxicity, leptomeningeal disease, and target location [NCT04030507 (8)]. Surgery and radiation are followed with traditional chemotherapeutic strategies to improve patient survival. However, standard breast cancer drugs, such as paclitaxel (Pax), doxorubicin (Doxil^©^), and trastuzumab, show poor efficacy in TNBC brain metastasis in clinical trials (NCT02260531, NCT01305941). The poor efficacy is partly attributed to the blood-brain barrier (BBB) and blood-tumor barrier (BTB), which prevent chemotherapies from accumulating within brain metastases at therapeutically effective concentrations (10–12).

The BBB consists of endothelial cells connected by tight junctions, basement membrane, pericytes, astrocyte foot processes, and microglia (10). The BBB efficiently regulates paracellular and transporter-mediated uptake of therapeutics within the brain. Efflux transporters expressed on BBB endothelial cells actively translocate therapeutics back into systemic circulation. Overall, the BBB is a physical and dynamic barrier to therapeutic entry into the brain.

Brain metastasis occurs when tumor cells from a peripheral tumor breach the BBB *via* diapedesis and disrupt paracellular connections at the BBB (10). When tumor cells colonize within the brain, the BBB evolves into the blood-tumor barrier (BTB), which allows tumor cell invasion, migration, and proliferation (10). Individual tumor cells can then grow past the BTB endothelia, co-opt the existing vasculature, and initiate angiogenesis to meet the increased need for nutrition and oxygen. Remodeled and newly formed capillaries are often distended, tortuous, and have atypical growth patterns (13). Despite the leaky vasculature, the BTB effectively limits permeation of most chemotherapeutics to subtherapeutic levels (14, 15). In contrast to high-grade primary brain tumors like glioblastoma, brain metastases show relatively low vascular density (11, 16), high intra-tumoral heterogeneity, and no correlation between vascular permeability and lesion size in preclinical models (10, 11). Recently, methods to improve drug delivery to brain metastases include nanoparticles, convective-enhanced delivery, and BTB disruption techniques such as low-intensity focused ultrasound (LiFUS).

LiFUS is a non-invasive approach in which ultrasound waves are combined with intravenously delivered microbubbles to disrupt the BBB/BTB. This technique has demonstrated successful preclinical and clinical opening of the BTB in glioblastoma models (17). However, there is little information regarding the efficacy of combinatorial LiFUS and chemotherapy in preclinical TNBC brain metastasis. Herein, we hypothesize that increasing vascular permeability within the tumor metastases by LiFUS with concurrent administration of Paclitaxel and Doxil will increase survival and efficacy in a preclinical model of TNBC brain metastasis. First, we quantified BTB disruption by the increased penetration of three drug markers, ^14^C-AIB (125 Da), Texas Red 3kDa (TxRed), and a 10kD dextran using a clinical LiFUS disruption device (ExAblate 3000, Haifa, Israel). We then evaluated the tumor progression and survival of LiFUS with standard-of-care combinatorial chemotherapy in a hematogenous preclinical metastasis model of MDA-MB-231Br TNBC. Finally, we studied the effects of combinatorial LiFUS and chemotherapy on the size and number of tumors within the brain.

## Methods and materials

### Chemicals and reagents

The fluorescent tracers TxRed 3kDa and Cs Blue 10kD were purchased from Invitrogen (Waltham, MA). 14C-Aminoisobutyric acid was obtained from American Radiolabeled Chemicals (ARC) (St. Louis, MO). Fetal Bovine Serum, phosphate-buffered saline (PBS) and Dulbecco’s modified eagle medium were obtained from Gibco™-Thermo Fisher Scientific (Waltham, MA). Paclitaxel was purchased from Selleck Chemicals (Houston, TX). Liposomal Doxil was kindly donated by West Virginia University Hospital Department of Pharmacy. IVISbrite D-Luciferin Potassium Salt Bioluminescent Substrate was obtained from PerkinElmer (Waltham, MA). Cremophore EL was purchased from Sigma-Aldrich (St. Louis, MO). All reagents and chemicals used were analytical grade.

### Animals

All experiments were approved by the Institutional Animal Care and Use Committee of West Virginia University. Athymic female Nu/Nu mice were purchased from the Jackson Laboratory (JAX^®^, Bar Harbor, ME, USA). All mice were approximately 25 g and 4-6 weeks old at the start of experimentation. Mice were anesthetized using 1.5-2% isoflurane. A custom animal restraint platform was constructed for repeatable placement and adjustment of mice on the FDA-approved ExAblate transducer, as outlined previously (18). The animals were placed on a rodent bed with an anesthesia mask. The baseplate was placed on the transducer, allowing positioning of the mouse in a supine position, with the skull immersed in degassed water. Vertical and horizontal adjustments were possible for tumor bearing mice based on animal size and weight. A single-channel MRI loop coil was fixed to the restraint for imaging. Animal body temperature during imaging and treatments was maintained using heating pads, and animals were monitored for any signs of distress.

### LiFUS experimental design

LiFUS experiments were conducted using the clinical ExAblate Neuro ultrasound technology (InSightec, Haifa, Israel). The bowl configuration of the transducer (1024 elements, 230 kHz) was coupled with a 3T clinical MRI scanner (Siemens MAGNETOM Prisma). To localize the target area, a T1-weighted image was acquired before sonication. The T1 Weighted Turbo Spin Echo (TSE) sequence variant had an FOV of 70 mm in the sagittal plane and was reformatted for targeting into axial and coronal planes. The repetition time (TR) was 700 msec, echo time (TE) was 7.5 msec, slice thickness was 0.7 mm, frequency and phase encoding matrix = 128 x 128 giving a voxel size of 0.5 mm x 0.5 mm x 0.7 mm, echo train length was 44 (acceleration factor used in TSE analysis for faster scan acquisitions), bandwidth = 434 Hz, Number of Excitations (NEX/Average) = 4 and total acquisition time was 3:39. Post-contrast imaging and local macro-hemorrhage sensitive Gradient Echo sequences were performed on some groups with FOV = 200 mm to acquire multi-slices in sagittal, axial, and coronal planes. The TR was 7.5 msec, TE = 3.7 msec, slice thickness = 7 mm, frequency and phase encoding matrix = 256 x 233 giving a voxel size of 0.4 mm x 0.4 mm x 7.0 mm and total acquisition time was 1:10. After MRI imaging was complete, targets were localized at the tumor site.

### Cell culture and *in vitro* cytotoxicity

The brain seeding cells of human triple-negative breast cancer transfected with GFP and Luciferase (MDA-MB-231Br) were donated as a gift by Dr. Patricia Steeg at the NIH. Cells were cultured in DMEM supplemented with 10% FBS and 2% Antibiotic-Antimycotic solution and were maintained at 37° C with 5% CO_2_.

An MTT (3-(4, 5-dimethylthiazol-2-yl)-2, 5-diphenyltetrazolium bromide) assay was used to evaluate cell viability. Briefly, MDA-MB-231Br or human brain endothelial cells (HBEC) were plated in a 96-well plate at a concentration of 1,000 cells per 100uL. After overnight incubation at 37°C to allow attachment, cells were treated with various concentrations of Pax, Doxil or a combination of the two drugs (n=4). Cells with media were used as negative control. Cell viability was determined at 72 and 96 hours using 10µL of MTT solution at a concentration of 5 mg/mL. After a 3 hour incubation at 37°C, MTT was removed and DMSO was added. Cell viability was determined by measuring the optical absorption of samples using Synergy2 multi-mode microplate reader (Biotek, Inc., Winooski, VT, USA) at 570 nm. Optical density value normalized to the blank negative control was used to calculate cell viability.

### Tumor initiation and bioluminescent imaging

Mice were anesthetized using 1.5-2% isoflurane and injected with MDA-MB-231-Br^GFP/Luc^ cells into the left cardiac ventricle at Day 0 followed by imaging using Bioluminescent imaging (BLI, IVIS Lumineer XV (PerkinElmer)). For all injections, cells were harvested at 70% confluency and injected at a concentration of 175,000 cells per mouse. Tumor burden was monitored biweekly using bioluminescence imaging. Mice were injected with D-luciferin potassium salt (150 mg/kg) dissolved in sterile PBS *via* intraperitoneal injection and then anesthetized under 1.5-2% isoflurane. Imaging was initiated 15 minutes post-injection to optimize the peak intensity based on luciferase/luciferin substrate kinetic profile. Darkfield images were acquired with an IVIS Lumineer XV (PerkinElmer) to detect peak intensity of the tumor signal. Regions of interest (ROIs) were drawn based on the cranial circumference using Living Image^®^ software (Xenogen, Alameda, CA, USA) and reported as radiance (photons/sec/cm^2^/steradian).

### Tracer studies and fluorescence quantification

Once neurological symptoms developed, the extent of LiFUS-mediated BTB opening was evaluated using differently sized tracers. ^14^C-Aminoisobutyric acid (AIB) (100 µCi/animal), TX Red (6 mg/kg) and 10 kDa Dextran (10 mg/kg) were administered intravenously and allowed to circulate for 10 mins post-injection. Brains were collected following circulation of the tracers and flash-frozen in isopentane at -70° C. Brains were sectioned using Leica CM3050 Cryostat (Leica Microsystems, Los Angeles, CA) to obtain 20 µm slices. Images were acquired using an upright Olympus MVX10 fluorescent stereomicroscope with Hamamatsu ORCA Flash4.0 v2 sCMOS camera fluorescence imaging. The optical zoom range was 0.63-12.6, N.A. = 0.5 with a DAPI/FITC/RFP/Cy5/Cy7 filters. Accumulation of TxRed and 10kD dextran was analyzed using RFP (588 nm) and DAPI (461 nm) channels respectively with overlay. CellSens image analysis software was used to determine permeability as sum intensity (SI) per area of brain for both fluorescent markers. Quantitative autoradiography was performed on the adjacent sections using autoradiography cassettes (GE Healthcare) with respective ^14^C standard values (0.1-862 nCi/g). Slides were developed for 21 days after a phosphor screen was placed over the samples (Fujifilm Life Sciences, 20 x 40 super-resolution). Analysis was performed using a high-resolution phosphor imager (FUJI FLA-7000, Fujifilm Life Sciences). ^14^C-AIB permeability was assessed using MCID Analysis (InterFocus Imaging LTD).

### Evaluation of *in vivo* efficacy and survival

Brain metastases were allowed to develop for 21 days followed by randomization and initiation of LiFUS and chemotherapy treatments. Mice were randomized into groups receiving Vehicle, LiFUS only, Pax (10mg/kg I.V, once a week), Doxil (5.6 mg/kg I.V, once a week), combined chemotherapy and LiFUS plus chemotherapy groups. Vehicle consisting of 1:1 blend of Ethanol and Cremophor EL diluted in saline (one part blend in nine parts saline) was administered to the control group. Pax was also formulated in the vehicle blend. Mice receiving LiFUS were sonicated once a week for three consecutive weeks at 0.3 cavitation dose for 60 secs. Chemotherapy and microbubbles (Definity^©^) were administered immediately before sonication. Mice were monitored routinely and collected when neurological symptoms developed.

### Data analysis

GraphPad^®^ Prism 6.0 (San Diego, CA) was utilized to determine differences in permeability, BLI and weights using ANOVA and t-test followed by Bonferonni’s multiple comparisons test. Survival data was analyzed using a Kaplan-Meier curve followed by determination of a log-rank value and Bonferroni-correction. All data represent mean ± SEM unless otherwise stated. Data was considered significant at p < 0.01 level.

## Results

### LiFUS increases permeability of TNBC metastatic brain lesions

**Figure 1:**
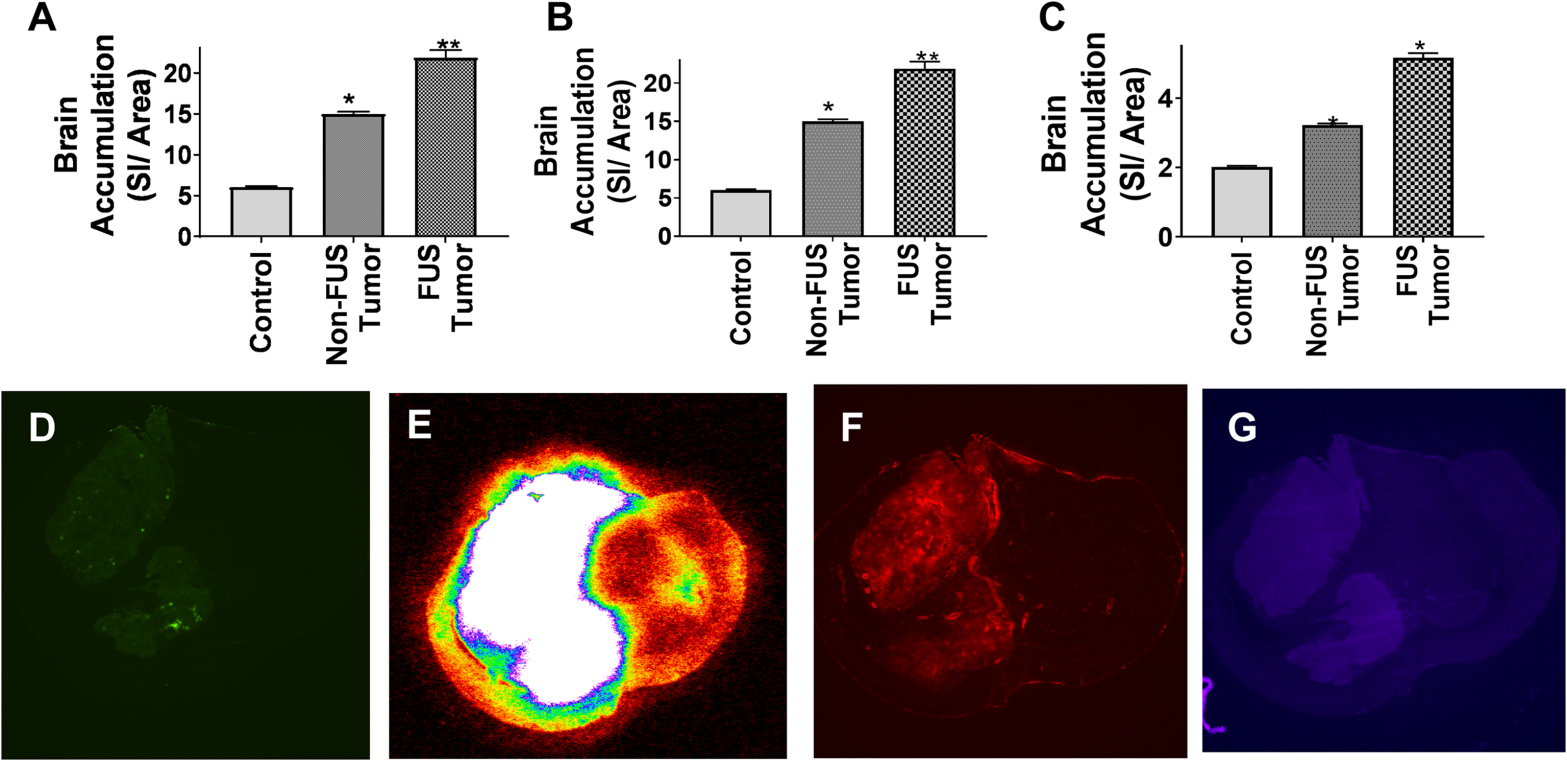
Permeability of metastatic brain lesions increases following LIFU mediated disruption. **(A-C)** BBB disruption with LIFU shows significantly higher accumulation of dye when the tumor region is disrupted by LIFU for AIB, TxRed and Cs Blue respectively. (p<0.0001) **(D)** GFP labeled tumor outline by fluorescent imaging. **(E-G)** BBB disruption by LIFU visualized within tumor region by autoradiographic and fluorescent imaging for AIB, TxRed and Cs Blue respectively. * p<0.01, ** p<0.001.

Mice with MDA-MB-231Br brain metastases were given three tracers of varying sizes followed by LiFUS treatment to assess how LiFUS-mediated BTB opening changes tumor vasculature. Tracer uptake within tumor lesions was found to be size-dependent with highest accumulation observed for small molecule ^14^C-AIB (~105Da) > TxRed 3kD > 10kD dextran. Accumulation of ^14^C-AIB within sonicated tumor lesions (604.9 ± 150nCi/g) was significantly higher (p<0.01) than the amounts found in contralateral non-sonicated tumors (76.3 ± 20.9 nCi/g) and control tumors (30.4 ± 10.6 nCi/g) **(** **Figure 1A** **)**. Similarly, TxRed uptake in the sonicated tumors was 18.9 ± 3.1 SI/area, which was significantly higher than non-sonicated contralateral tumors and control tumors, which were 15 ± 0.6 and 8.1 ± 3.2 SI/area respectively (p<0.01) **(** **Figure 1B** **)**. Lastly, the lowest overall uptake was observed for the 10kD dextran in sonicated lesions with 5.6 ± 0.13 kDa, meanwhile non-sonicated contralateral tumors and control tumors accumulated at 4.22 ± 0.05 SI/area and 4.32 ± 0.34 SI/area respectively (p<0.01) **(** **Figures 1C–G** **)**.

### Differential permeability between tumor core and periphery in LiFUS-disrupted tumors

**Figure 2:**
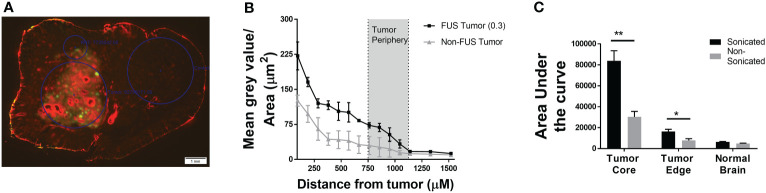
Differential permeability of TxRed (3kDa) is observed between tumor core and periphery in LIFU-disrupted tumors. **(A)** Fluorescent image showing TxRed accumulation within tumor (GFP). **(B)** Quantitative differential accumulation of TxREd between tumor core, edge and adjacent healthy brain. **(C)** Area under the curve for extent of brain accumulation of TxRed for non-sonicated and sonicated tumors and healthy brain following LIFU mediated BTB disruption. (p<0.001). * p<0.01, ** p<0.001.

We evaluated regional diffusion inside lesions for small-to-moderate sized chemotherapies after disruption by LiFUS using the TxRed 3kDa as an upper limit marker. For various sonicated metastases, permeation of TxRed 3kDa relative to distance was plotted between normal brain, tumor edge, and tumor core as previously reported (19). When compared to non-sonicated tumors, the mean permeability of TxRed 3kDa increased 2.6 to 3.1-fold in the tumor core and 1.9 to 2.3-fold in the tumor edge **(** **Figures 2A, B** **)**. There was no significant difference in TxRed 3kDa uptake within the normal brain around sonicated tumor compared to a non-sonicated tumor **(** **Figure 2C** **)**.

### MDA-MB-231Br cells are more sensitive to concurrent Pax+ Doxil treatment compared to individual drug therapy

**Figure 3:**
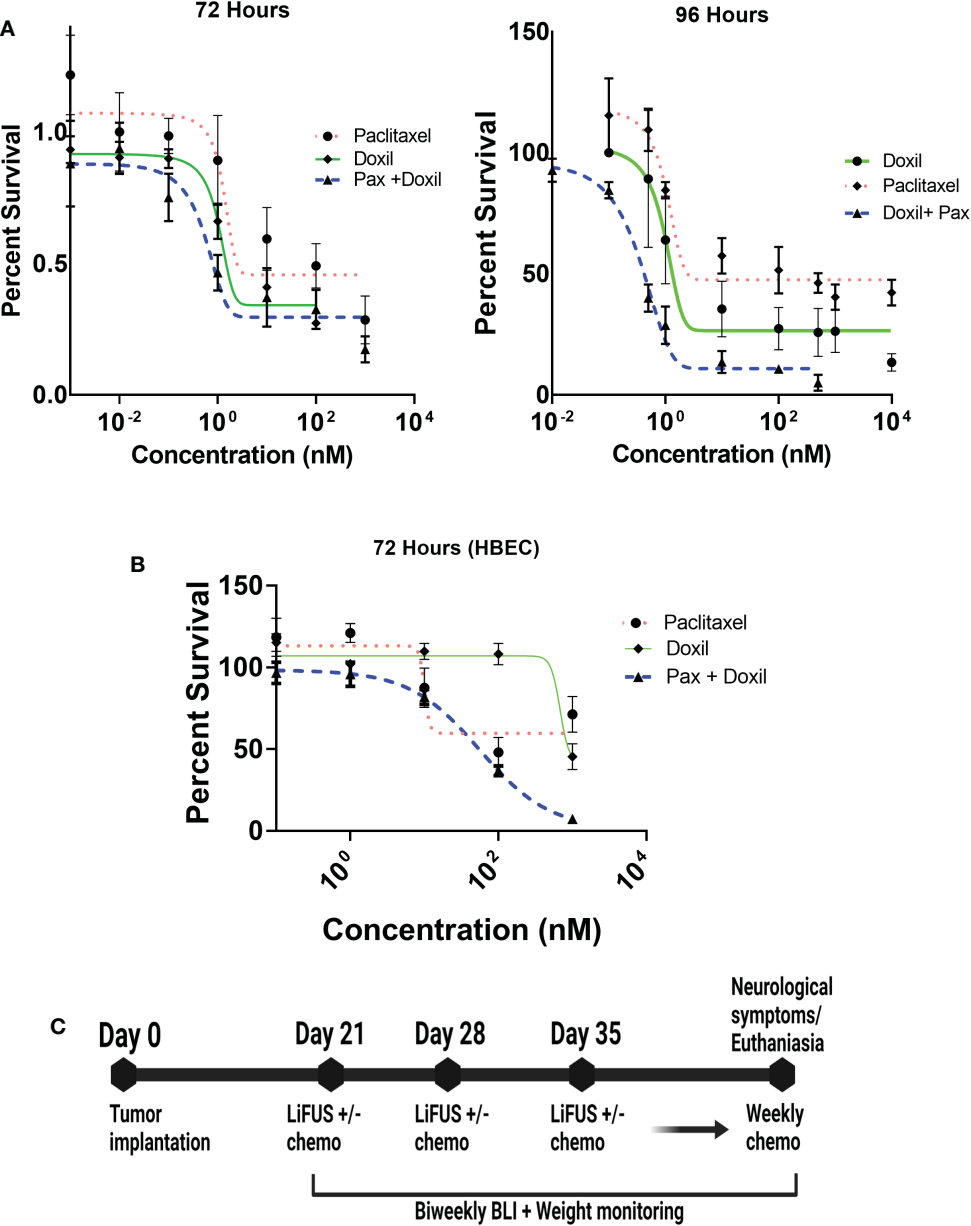
Treatment strategy for LIFU+ combinatorial chemotherapy. **(A)** Paclitaxel+ Doxil have additive action against MDA-MB-231Br cells (IC50 1.8± 0.91nM) **(B)** Paclitaxel+ Doxil are safe at the administered concentrations to HBEC cells. (IC50 56 ± 1.7nM) **(C)** All animals were treated with LIFU or chemotherapy for one session, every week for three weeks, after which weekly treatment with chemotherapy was continued until collection.

Anti-cancer activity of the combinatorial therapy was evaluated in MDA-MB-231Br or HBEC *in vitro* using MTT assays. The IC_50_ values for Pax and Doxil were 20.08 ± 2.43 nM and 10.2 ± 1.2 nM respectively at 72 hours, and 9.6 ± 3.9 nM and 8.1 ± 0.5 respectively at 96 hours **(** **Figure 3A** **)**. Treatment with Pax+Doxil showed an additive effect in inhibiting growth of MDA-MB-231Br with an IC_50_ of 1.8 ± 0.32 nM at 72 hours and 0.4 ± 0.08 nM at 96 hours (p<0.01) **(** **Figure 3A** **)**. Combinatorial treatment with Pax+Doxil was found to be safe at the administered concentrations in HBEC cells with an IC_50_ of 56 ± 1.7 nM at 72 hours (**Figure 3B**).

### Concurrent LiFUS with Doxil significantly increases efficacy and slows tumor progression in mice bearing MDA-MB-231Br brain metastasis

**Figure 4:**
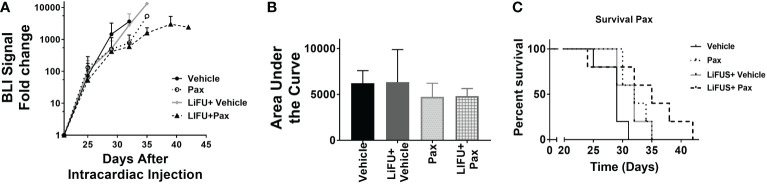
Concurrent therapy of LIFU with paclitaxel does not decrease tumor burden or increase survival in a preclinical model of brain metastasis of breast cancer. **(A)** BLI signal versus time in mice with treatment beginning on day 21 (n=5-7). Mice treated with 10mg/kg Pax **(B)** Area under curve **(C)** Kaplan-Meier survival plot of mice starting 21 days after intracardiac injection of MDA-MB-231Br breast cancer cells.

**Figure 5:**
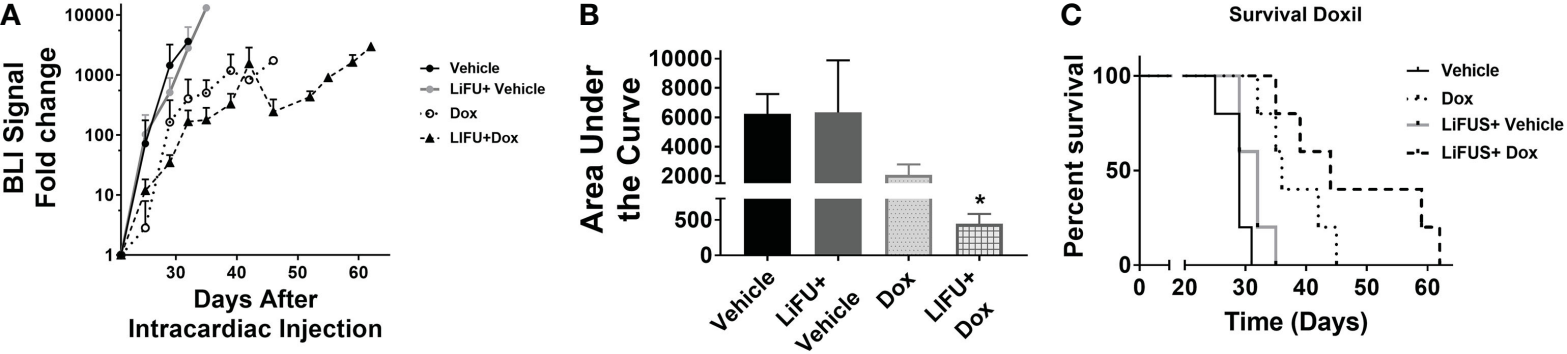
Concurrent LIFU+ standard of care Doxil decreases tumor burden and increases survival non-significantly. **(A)** BLI signal versus time in mice with treatment beginning on day 21. **(B)** Area under curve for representative tumor burden shows no significant difference (n=5-7) **(C)** Kaplan-Meier survival plot of mice starting 21 days after intracardiac injection of MDA-MB-231Br breast cancer cells. * p<0.01.

Next, efficacy of LiFUS, individual drugs and their combination was evaluated *in vivo* in our preclinical model of TNBC brain metastases. Mice inoculated with MDA-MB-231Br through intracardiac injections were treated with either: a hydrophobic molecule with broad-spectrum anti-neoplastic activity (Pax), a long acting nano-particulate formulation of a hydrophobic drug (Doxil) or a combination of these w/w.o LiFUS **(** **Figure 3C** **)**. There was no significant improvement in survival or reduction in tumor burden in groups receiving LiFUS only, Pax only, or LiFUS+Pax compared to control **(** **Figures 4A, B** **)**. The median survival for control, LiFUS only, Pax only and LiFUS+Pax was 29, 32, 32 and 35 days respectively **(** **Figure 4C** **)**. Compared to vehicle and LiFUS + vehicle groups, treatment with LiFUS+ Doxil significantly reduced tumor burden when compared at day 32 **(** **Figures 5A, B** **)**. Treatment with Doxil and LiFUS+ Doxil also significantly improved survival to 36 and 44 days respectively compared to vehicle (p<0.01) **(** **Figure 5C** **)**.

**Figure 6:**
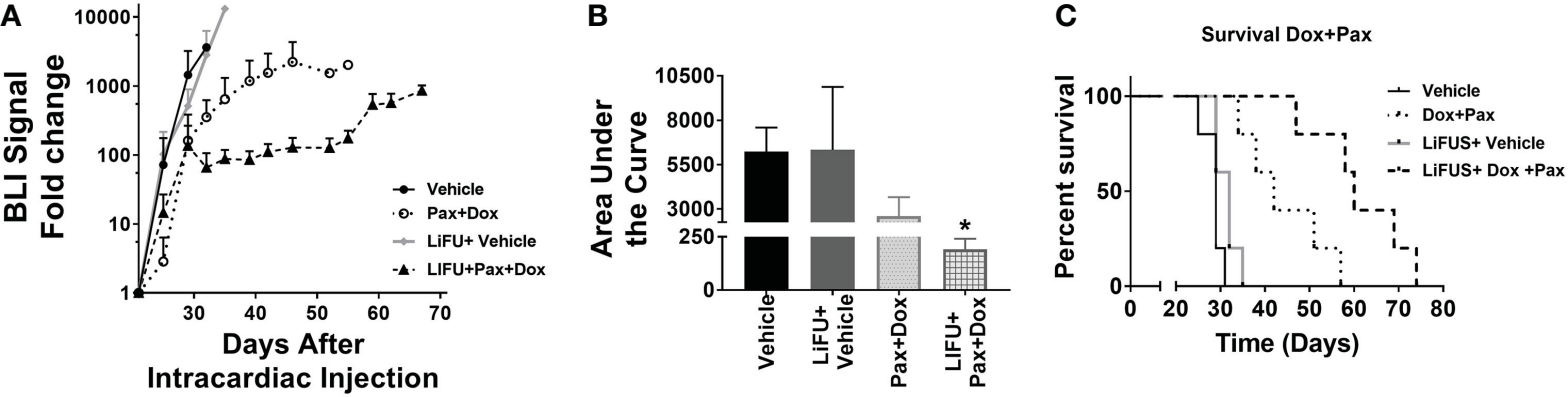
Concurrent therapy of LIFU with combination therapy of paclitaxel and Dox-NP decreases tumor burden and increases survival significantly in a preclinical model of brain metastasis of breast cancer. **(A)** BLI signal versus time in mice with treatment beginning on day 21 (n=5-7). Mice treated with 10mg/kg Pax or 5.6mg/kg Liposomal Dox **(B)** Area under curve **(C)** Kaplan-Meier survival plot of mice starting 21 days after intracardiac injection of MDA-MB-231Br breast cancer cells. * p<0.01.

An additive action was observed for LiFUS+Pax+Doxil combinatorial group with a median survival of 60 days, which was significantly higher than control, LiFUS only and the Pax+Doxil (42days) groups (p<0.01). The LiFUS+Pax+Doxil group showed the lowest tumor burden **(** **Figure 6A** **)** compared to all other groups at day 32, indicating slower tumor progression (**Figures 6B, C**).

**Figure 7:**
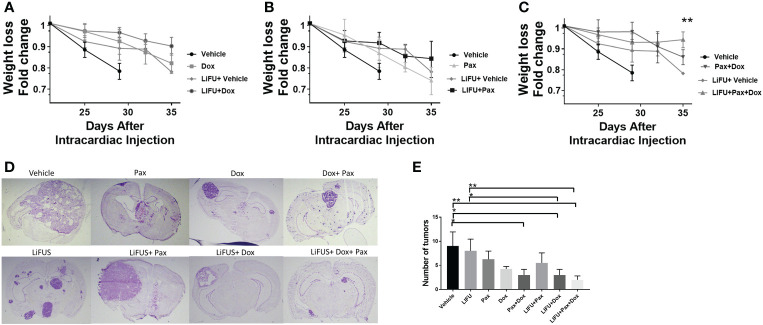
LIFU plus combinatorial chemotherapy therapy maintains weight in mice with metastatic MDA-MB-231Br tumors: **(A-C)** Weights loss for mice treated with LIFU+Dox-NP+Pax was significantly lower than the weight loss observed for vehicle or LIFU+vehicle. While LIFU+Pax, LIFU+ Dox-NP reduced weight loss compared to vehicle, the observed difference was non-significant. **(D, E)** Tumor count and imaging demonstrate LIFU+ combinatorial chemotherapy reduce number of brain metastases. * p<0.01, ** p<0.001.

Lastly, we evaluated the effect of LiFUS with combinatorial Pax+Doxil on weight loss and number of metastatic lesions within the brain. Individual drug therapy and LiFUS alone had little effect on weight loss or the number of metastatic lesions, however LiFUS+Pax+Doxil significantly decreased both when compared to a vehicle control **(** **Figures 7A–E** **)**.

## Discussion

Despite the emergence of new therapies, disease prognosis and overall survival of patients suffering from TNBC brain metastasis remains poor. Recent research suggests non-invasive BBB disruption by LiFUS can potentially improve therapeutic outcomes in neurological conditions like Alzheimer’s and Parkinson’s disease. However, further research is needed on these non-standardized instruments and dosing regimens to close disparities found in various studies before implementation in clinical practice. In this study, we demonstrate that a clinically relevant LiFUS regimen significantly increases the therapeutic efficacy of Pax and Doxil in a preclinical model of TNBC brain metastasis through BTB disruption.

To investigate the effects of LiFUS on tumor vasculature, we first quantified the size dependency of BTB opening in animals with TNBC brain metastases using tracers. In high-grade gliomas, changes in tumor permeability following LiFUS is characterized in previous clinical and preclinical reports (20–23). However, compared to high-grade gliomas, the BTB in brain metastasis is anatomically different and has a lower vascular density and smaller perforations (pores) within blood vessels (11, 24). Hence, it is crucial to identify the degree of LiFUS-mediated BTB disruption within brain metastases of breast cancer, which has high intratumoral heterogeneity. Previous research using LiFUS in brain metastasis models revealed a knowledge gap regarding partial responses by animals in LiFUS treatment groups, which could not be explained solely by contrast enhancement (25, 26). Thus, there is a need for supplemental quantitative analysis to MRI. Our data indicates BTB permeability in TNBC brain metastasis is highly size-dependent following LiFUS-mediated disruption (^14-^C-AIB>TxRed> 10kD Dextran). Quantitative fluorescence microscopy was used to evaluate diffusion of a small-molecule drug sized tracer (TxRed) between the tumor core and healthy brain tissue within sonicated lesions. We found differential tumor uptake between the tumor core and healthy brain with maximal tracer accumulation within tumor core and periphery. Taken together, our data suggest LiFUS alters BTB permeability in TNBC metastasis in a size-dependent manner, with higher permeation of tracers within the sonicated tumor core region.

Next, we determined whether LiFUS-mediated enhancement of BTB permeability affected the therapeutic outcomes of TNBC metastases. Pax is routinely used to treat peripheral breast cancer (27). However, due to its pharmacokinetic properties, it has low brain distribution and accumulation. Pax is poorly water soluble (logP 3), which correlates with inadequate passive and paracellular permeability into the brain and brain metastasis (28). Thus, we evaluated the effect of LiFUS on efficacy of a small hydrophobic chemotherapeutic like Pax for the treatment of TNBC brain metastasis. In mice with MDA-MB231Br brain metastases, the combination of LiFUS and Pax exhibited a trend of decreased tumor development and improved survival; however, this trend was not statistically significant. While LiFUS may have increased the brain’s permeability to Pax, efflux transporters like P-gp and BCRP expressed on the BBB and BTB likely prevent its accumulation. Recent *in vitro* studies support our findings, showing that ultrasound therapy at 150 kHz can promote polymerization of Pax stabilized microtubules, partially counteracting Pax’s cytotoxic effects on breast and ovarian cancer cells (29). A change in the frequency used can potentially neutralize this effect. Additionally, recent research indicates LiFUS downregulates expression of efflux transporters like P-gp at the BBB approximately 10-72 hours after exposure (17, 30). Timed administration of small hydrophobic chemotherapeutics within windows of transporter downregulation after LiFUS could potentially increase the efficacy by making the substrates less prone to efflux. This is especially true for permeability-restricted, metastatic endocrine-resistant breast cancers such as TNBC (31). LiFUS with combined Pax+ resistance-specific mechanistic treatments (e.g., PDL1 antibody like Atezolizumab) could be extremely beneficial to increase permeation in such cases. Future research is necessary to understand the effects of LiFUS-assisted combinatorial small-molecule and endocrine resistance tailored therapy and its downstream outcomes.

Nanoparticles offer numerous advantages like protection of loaded therapeutic molecules as a carrier, sustained drug release, prolonged drug residence time, and improved tumor targeting. A previous study by Aryal et al. demonstrated LiFUS allowed accumulation of clinically efficacious concentrations of liposomal doxorubicin within the brains of glioma-bearing rats (32). In our next set of experiments, we investigated how LiFUS-mediated BTB disruption affects the efficacy and survival of mice with TNBC brain metastases with concurrent administration of a nano-particulate system with long systemic circulation. Our results demonstrate LiFUS+ Doxil significantly increases survival and slows tumor progression in mice with TNBC brain metastases. Our findings imply liposomal formulations of free drugs may entrap the drug or nano-particulate system within the disrupted barrier, thereby prolonging its action on tumor lesions. Further, sustained drug release and PEGylation-mediated decrease in phagocytic uptake may allow for prolonged activity of doxorubicin within tumor lesions. Future studies may explore a targeted single sustained lipid delivery formulation to enhance drug accumulation and efficacy within tumor lesions in combination with LiFUS.

Finally, we explored a dual-chemotherapeutic strategy as a multimodal approach to treat TNBC brain metastasis. In our *in vitro* studies, we observed a significantly lower IC_50_ for MDA-MB-231Br cells when treated with combinatorial Pax and Doxil compared to chemotherapy alone, suggesting synergism in tumor inhibition by two unique mechanisms. In our preclinical model of TNBC brain metastasis, administration of Pax-Doxil in combination with LiFUS significantly inhibited tumor progression when compared to control and individual drug chemotherapy groups. Furthermore, mice receiving Pax+ Doxil with LiFUS had significantly improved overall survival as compared to control and Pax+ Doxil groups. Concurrent LiFUS with combinatorial Pax+ Doxil group had the least tumor-associated weight loss. Weight loss is a component of cancer cachexia, and it exacerbates functional impairment and skeletal muscle loss (33). The reduction in cancer-related weight loss, reduced number of brain metastases and targeted BTB opening observed in this study may potentially mitigate the treatment-associated decrease in the patient’s quality of life.

This research successfully indicates that combining LiFUS with Pax and Doxil improves overall survival and reduces tumor progression in a preclinical model of TNBC brain metastases. While our study demonstrates increased therapeutic efficacy of combinatorial chemotherapy with LiFUS, there are a few limitations. First, we conducted a limited histological analysis of tumor tissue following LiFUS therapy. A more thorough investigation is required, particularly to identify changes in tumor histology following several treatments. While our investigation measured tumor permeability at the conclusion of the survival study using TxRed, future reports can expand on this work by visualizing drug uptake within lesions during sonication treatments. The effects of combined immunotherapy and small-molecule chemotherapy must also be assessed using LiFUS. This is crucial, especially considering repeated BBB opening by LiFUS can activate inflammatory pathways and alter immune responses (34, 35). Previous research on primary and metastatic brain tumors has demonstrated that immune responses to LiFUS-mediated opening can be relatively mild and may/may not interfere with co-administered immune adjuvants (36, 37).

Lastly, clinical translation of this technique would require investigating molecular and physicochemical alterations post stereotactic or whole-brain radiation. Since radiation is the first line of treatment for most patients with brain metastases, the effect of LiFUS on an irradiated brain and the potential to combine LiFUS and radiation mediated therapy to increase drug delivery need to be extensively explored. This study lays the groundwork for LiFUS-mediated multimodal therapeutic delivery in brain metastases of breast cancer.

## Conclusion

LiFUS-induced transient disruption of the BBB is emerging as a treatment modality to combat low drug brain penetration and retention, as well as systemic toxicity of intravenously administered therapy. Our study offers a foundation for creating therapeutic strategies intended to maximize uptake and efficacy of drug combinations in TNBC brain metastases using LiFUS. Our tracer work also highlights the necessity to further study potential harm to normal brain with increased LiFUS-mediated penetration of drugs within the tumor lesions. Further research may benefit by combining other permeability-enhancing techniques such as radiation and endovascular microcatheter administration with LiFUS (38, 39). In conclusion, we demonstrate that employing LiFUS to administer combined Pax and Doxil to TNBC brain metastases improves overall survival and slows metastatic progression in our preclinical model.

## Data availability statement

The original contributions presented in the study are included in the article/supplementary material. Further inquiries can be directed to the corresponding author.

## Ethics statement

The animal study was reviewed and approved by The Institutional Animal Care and Use Committee of West Virginia University.

## Author contributions

All authors had full access to the data in the study and took responsibility for the integrity of the data and the accuracy of the data analysis. Conceptualization: TA, AR, and PL; methodology: TA, PL, and JC; investigation: TA, SS, KB, CW, RF, BK, TP, PW, OW, JC, VF, and PL; formal analysis: TA and PL; resources: TA, PL, and AR; writing, review and editing: TA, SS, KB, RF, CW, BK, TP, PW, OW, JC, VF, and PL; supervision: PL and AR; funding acquisition: TA and PL. All authors contributed to the article and approved the submitted version.
